# An adapted transdiagnostic sleep and circadian intervention for adults with excess weight and suboptimal sleep health: pilot study results

**DOI:** 10.1093/sleepadvances/zpae037

**Published:** 2024-06-13

**Authors:** Christopher C Imes, Christopher E Kline, Sanjay R Patel, Susan M Sereika, Daniel J Buysse, Allison G Harvey, Lora E Burke

**Affiliations:** School of Nursing, University of Pittsburgh, Pittsburgh, PA, USA; Department of Health and Human Development, University of Pittsburgh, Pittsburgh, PA, USA; Center for Sleep and Cardiovascular Outcomes Research, University of Pittsburgh, Pittsburgh, PA, USA; School of Nursing, University of Pittsburgh, Pittsburgh, PA, USA; Department of Psychiatry, University of Pittsburgh, Pittsburgh, PA, USA; Department of Psychology, University of California, Berkeley, Berkeley, CA, USA; School of Nursing, University of Pittsburgh, Pittsburgh, PA, USA

**Keywords:** actigraphy, behavioral sleep medicine, circadian rhythms, obesity

## Abstract

**Study Objectives:**

This single-arm, mixed-methods, pilot study examined the feasibility and preliminary efficacy of an adapted version of the transdiagnostic intervention for sleep and circadian dysfunction (TranS-C) on multidimensional sleep health (MDSH) in a sample of adults with excess weight and suboptimal sleep health.

**Methods:**

Participants received up to eight, weekly, remotely delivered, tailored TranS-C sessions. At pre- and post-intervention, the Pittsburgh Sleep Quality Index, Epworth Sleepiness Scale, and 7 days of Fitbit data were used to evaluate changes in sleep dimensions (regularity, alertness, timing, satisfaction, duration, and efficiency) and the composite MDSH score. Study feasibility examined recruitment, data collection, and intervention engagement (completion of core TranS-C sessions). Acceptability of the intervention was assessed with semi-structured interviews, which were analyzed using thematic analysis.

**Results:**

From 85 referrals, 11 individuals were eligible, and 10 completed the study. All intervention participants completed the measures needed to calculate their composite MDSH score and completed the core intervention sessions. Themes from interviews support the intervention’s remote delivery approach, applicability of the information provided, and impact on self-reported health. The intervention resulted in a large improvement in the mean composite MDSH score (Cohen’s *d* = 1.17). Small-to-large effects were also observed for individual sleep health dimensions except for timing.

**Conclusions:**

Adapted TranS-C is acceptable for adults with excess weight and suboptimal sleep health and may be effective at improving short-term MDSH. With changes to recruitment methods, a larger study is feasible. Limitations include the small sample size and the lack of a control condition.

**Statement of Significance**

The Transdiagnostic Intervention for Sleep and Circadian Dysfunction (TranS-C) adapted for adults with excess weight and suboptimal sleep health was feasible, acceptable, and improved composite multidimensional sleep health scores and individual sleep dimensions. Future work should refine the novel intervention and examine if improving multidimensional sleep health through the TranS-C in the context of a behavioral weight loss intervention improves weight outcomes.

The increasingly high prevalence of obesity represents a public health crisis in the United States. Currently, obesity (body mass index [BMI] of ≥ 30 kg/m^2^) affects 41.9% of adults and is projected to reach 48.9% by 2030 [1, 2]. Obesity is associated with an increased risk for cardiometabolic disease and multiple cancers. The increase in the prevalence of obesity has been paralleled by an increased prevalence of poor sleep health. From 2004 to 2017, the percentage of adults who obtained ≤6 hours of sleep in a 24-hour period increased from 28.6% to 32.9% [3]. Cohort and epidemiological studies also show that approximately 40% of the US population experiences poor sleep quality [4, 5]. Poor sleep health increases the risk for excess weight, obesity, and cardiometabolic disease through behavioral (e.g. increased energy intake, less motivation to exercise) and physiological (e.g. impaired glucose tolerance, altered hormone levels) mechanisms [6–10]. Other dimensions of sleep (timing, efficiency, and regularity) are also related to weight changes and obesity [11–13]. However, prior attempts at using a sleep-focused intervention to improve weight outcomes have been disappointing due, in large part, to a failure to successfully improve sleep [14, 15]. Interventions that are effective at improving sleep in an adult population with overweight/obesity are sorely needed to leverage sleep as a novel pathway for addressing the obesity epidemic.

The multidimensional sleep health (MDSH) framework recognizes that good sleep promotes health beyond the simple absence of sleep disorders [7]. Dimensions commonly incorporated into MDSH definitions include regularity, satisfaction, alertness, timing, efficiency, and duration [7]. The Transdiagnostic Intervention for Sleep and Circadian Dysfunction (TranS-C) is grounded in sleep and circadian principles with the goal of optimizing MDSH dimensions through altering psychosocial, behavioral, and cognitive processes [16]. In a study of 176 youths, TranS-C improved weeknight to weekend discrepancy in wake-up time (regularity), sleep duration, sleep quality (satisfaction), and sleepiness (alertness) compared to an educational control (effect sizes = 0.29 to 0.55) [17]. In a randomized controlled trial of TranS-C among 121 midlife and older adults with serious mental illness, TranS-C improved sleep efficiency, wake time variability, and a composite sleep health score compared to usual care at immediate post-intervention and 6-month follow-up [18]. Despite the emerging evidence supporting the efficacy of TranS-C and the link between poor sleep and obesity, the efficacy of TranS-C has not been evaluated in adults with obesity.

The aims of this single-arm pilot study were to: (1) examine the feasibility and acceptability of a remotely delivered TranS-C-based intervention in a sample of adults with excess weight and suboptimal sleep health interested in weight loss and (2) explore the preliminary efficacy of the intervention on composite MDSH score and individual sleep health dimensions.

## Methods

### Recruitment, screening, and baseline data collection

The primary recruitment source was a University-sponsored research registry. Interested individuals were referred to the research team for additional screening. Screening included a preliminary survey with a follow-up phone call. If eligible, informed consent was obtained at an in-person session and baseline data, including weight, was collected. Post-session, participants wore a home sleep apnea testing device (WatchPAT 300, Zoll Itamar, Atlanta GA) to screen for severe obstructive sleep apnea (apnea–hypopnea index [AHI] > 30 events/hour) and wore a Fitbit, while completing a daily sleep diary, for 7 days. To examine the feasibility of TranS-C among adults interested in weight loss, the inclusion and exclusion criteria mirrored that of a behavioral weight loss intervention. Inclusion criteria included: (1) age ≥ 18 years, (2) BMI > 27 and ≤ 43 kg/m^2^, and (3) poor sleep health on ≥ 1 MDSH dimension. Exclusion criteria included: (1) current treatment for a serious mental illness or sleep disorder; (2) diagnosis of obstructive sleep apnea or AHI > 30 events/hour based on home testing; (3) current shift work; (4) history of bariatric surgery, current use of a weight loss medication/participation in a weight loss program, or loss of ≥ 5% weight in the past 6 months; (5) other household member enrolled in the study; and (6) presence of severe binge eating (score > 32 on the Binge Eating Scale) [19]. The study was approved by the University of Pittsburgh Human Research Protection Office and registered at ClinicalTrials.gov (NCT04990206).

### Procedures

MDSH was assessed at baseline (pre-intervention) and post-intervention using the approach developed by Kline et al. that evaluates sleep health along six dimensions, regularity, satisfaction, alertness, timing, efficiency, and duration [20]. The dimension of satisfaction was calculated using the sleep quality item from the Pittsburgh Sleep Quality Index (PSQI) [21]. Alertness was calculated using the total score from the Epworth Sleepiness Scale (ESS) [22]. Participants wore a Fitbit Charge 4 for 7 days while completing an online version of the consensus sleep diary [23]. The dimensions of regularity (standard deviation of wake time), timing (mean sleep midpoint), efficiency, and duration were calculated using Fitbit data averaged across the 7 days. A minimum of 5 days of data, including at least one weekend night, was needed to calculate the dimensions. Each sleep dimension was dichotomized as either “good” or “poor” based on clinically and scientifically relevant cutoffs [20]. The number of “good” dimensions were summed for a composite MDSH score with higher values indicating better sleep health.

### TranS-C intervention and post-intervention data collection

TranS-C was delivered remotely by two-way video conferencing. Participants received up to eight, weekly, tailored sessions lasting approximately 50 minutes. The first four core sessions included: establishing regular sleep–wake times, developing a wake-up and wind-down routine, improving daytime functioning, and correcting unhelpful sleep-related beliefs. Sessions five and six included reducing sleep-related worry and negotiating sleep in a complicated environment. Session seven, focused on reducing nightmares, was only delivered when applicable. The last core session focused on behavioral maintenance (see Table 1 for details). Post-intervention data were collected approximately 1 week after the last session using the same approach as baseline data collection. Additionally, a semi-structured interview was conducted to examine the study’s acceptability.

**Table 1.** TranS-C Sessions

Session	Topic	Core session (yes/no)	All modules included
1	Establishing regular sleep–wake times	Yes	Case formulation, sleep and circadian education, behavioral change and motivation, and goal setting
2	Learning a wake-up and wind-down routine	Yes	
3	Improving daytime functioning	Yes	
4	Correcting unhelpful sleep-related beliefs	Yes	
5	Reducing sleep-related worry	No	
6	Negotiating sleep in a complicated environment	No	
7	Reducing nightmare (only delivered if applicable)	No	
8	Maintenance of behavior change	Yes	

### Operational definitions

To examine the feasibility of a larger trial, we examined the study’s recruitment, data collection, and engagement. For recruitment, we compiled the numbers of individuals who were referred, completed the preliminary screening, were eligible based on preliminary screening, completed the in-person session and baseline data collection, and received the intervention and completed the post-intervention data collection. For data collection feasibility, we report completion rates of essential study measures, i.e. those needed to calculate the composite MDSH score (PSQI, ESS, and Fitbit data). Engagement was defined as the percentage of intervention participants who completed all of the core TranS-C sessions. Acceptability of the intervention was examined through qualitative themes in semi-structured interviews. Lastly, the preliminary efficacy of the intervention was determined by measuring pre–post changes in composite MDSH score and individual MDSH dimensions.

### Analysis

Study feasibility was examined using descriptive statistics. For the acceptability of the intervention, the semi-structured interviews were transcribed and analyzed using thematic analysis. Sociodemographic characteristics and MDSH data were described using means and standard deviations for continuous variables and frequencies and percentages for categorical variables. The individual sleep dimensions were summarized as the proportion of the sample who met the criteria for “good” sleep with a 95% confidence interval. Given the small sample size of this pilot study, the effect size of the intervention on composite MDSH score, individual MDSH dimensions, and weight were examined. Cohen’s *d* for repeated measures (*d*_rm_) was used to calculate the effect size for the continuous variables [24], whereas Cohen’s *d* of the pre-test post-test changes in proportions was calculated using the dichotomous variables.

## Results

**Figure 1.**
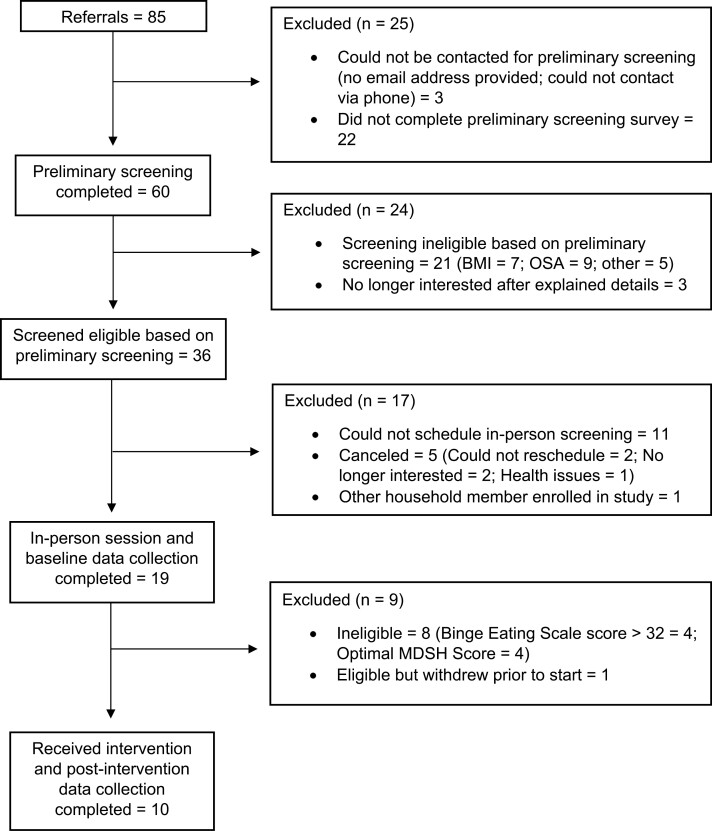
CONSORT participant flow diagram.

Participant flow is illustrated in Figure 1. Eighty-five individuals were referred from the University-sponsored research registry, of whom 60 (70.5%) completed the preliminary screening survey, 36 (51.4%) of these individuals met screening criteria, and 19 (52.8%) of these completed the in-person session. Eleven (57.9%) of these individuals were eligible for the study, but one withdrew prior to receiving the intervention. The remaining 10 participants completed the intervention. Study ineligibility (*n* = 29), interested individuals not completing the preliminary screening survey (*n* = 22), and the inability to contact or schedule individuals for them to complete required study procedures (*n* = 16) were the leading causes of attrition.

The 10 participants who received the intervention and completed the follow-up assessment were mostly female (*n* = 7) and white (*n* = 8) with a mean age of 49.0 ± 16.0 years and mean BMI of 33.5 ± 3.5 kg/m^2^. All participants completed ≥ 2 years of college and 50% had graduate school education. Six participants worked full time, two worked part time, one was a full-time student, and one was retired. All participants had health insurance and, while gross annual income ranged from $25 000 to over $100 000, all reported that their household income met basic needs. Seven participants received 7 TranS-C sessions and three received eight sessions. One hundred percent of the essential data were collected from participants who completed the intervention. Mean days of Fitbit wear days were 6.6 ± 0.7 and 6.8 ± 0.6 during the pre- and post-intervention collection phases, respectively. Furthermore, 100% of participants undergoing the intervention received all of its core components.

Overall, the intervention was acceptable for the participants. Positive themes regarding the intervention included the participants being “in favor of the remote [nature of the] intervention” with several commenting that they could not or would not have participated in the study if it was in person. The participants found the intervention to be “informative” and they were able to “develop a routine” that incorporated the core behavioral aspects of TranS-C. The intervention “improved multiple self-reported health aspects” including sleep, energy levels, and reduced stress. However, there were unfavorable responses regarding the duration of the intervention. Some session content was “repetitive” or “not applicable to the individual” and several participants suggested that the “intervention could be shortened” by a session or two.

The intervention had a large effect (Cohen’s *d*_rm_ = 1.17) on the mean composite MDSH score from pre- to post-intervention (3.80 ± 1.23 [range 2–5] vs. 4.90 ± 0.70 [range 4–6]). When examining the dichotomous outcomes (“poor” sleep vs. “good” sleep), small-to-large effects were seen in all dimensions (effect sizes ranging from 0.21 to 1.31) except for timing. At post-intervention, all participants had “good” sleep quality, alertness, and sleep efficiency. For the continuous variables, improvements were observed across all dimensions (effect sizes ranging from 0.29 to 0.90; Table 2). TranS-C had no effect on weight (effect size = 0.02; data not shown).

**Table 2.** Pre-intervention and Post-intervention Composite Multidimensional Sleep Health (MDSH) Score, Individual Dimensions, and Effect Sizes

Composite MDSH score (sum of dichotomous variables; higher scores indicate better sleep health)			
	Pre-intervention mean (SD)	Post-intervention mean (SD)	Cohen’s *d*_rm_
Composite Sleep Health Score	3.80 (1.23)	4.90 (0.74)	1.17

Sleep health dimensions (dichotomous variables)			
Dimension (operational definition of “good” sleep)	Pre-intervention Proportion of ‘good’ dimension (95% CI)^a^	Post-intervention Proportion of ‘good’ dimension (95% CI)^a^	Cohen’s d^b^
Regularity (SD of wake time ≤ 60 minutes)	0.30 (0.07 to 0.65)	0.40 (0.12 to 0.74)	0.21
Satisfaction (fairly/very good sleep quality)	0.40 (0.12 to 0.74)	1.00 (0.69 to 1.00)	1.31
Alertness (Epworth Sleepiness Scale score ≤ 10)	0.80 (0.44 to 0.97)	1.00 (0.69 to 1.00)	0.66
Timing (mean midpoint 2:00 and 4:00 a.m.)	0.60 (0.26 to 0.88)	0.60 (0.26 to 0.88)	0.00
Efficiency (mean sleep efficiency ≥ 85%)	0.90 (0.56 to 0.99)	1.00 (0.69 to 1.00)	0.45
Duration (mean total sleep time 6–8 hours)	0.70 (0.35 to 0.93)	0.90 (0.56 to 0.99)	0.50
*Sleep health dimensions (continuous variables)*			

Dimension	Pre-intervention Mean (SD)	Post-intervention Mean (SD)	Cohen’s *d*_rm_
Regularity, minutes^c^	85.9 (31.9)	73.5 (46.9)	−0.29
Satisfaction, 1–4^d^	2.2 (0.8)	3.0 (0.0)	0.88
Alertness, Epworth Sleepiness Scale score^c^	7.1 (3.7)	6.0 (3.1)	−0.37
Timing, minutes past midnight^c^	236 (73)^b^	198 (109)^b^	−0.63
Efficiency, %	88.6% (2.3%)	90.0% (2.6%)	0.90^e^
Duration, minutes	379 (57)	396 (39)	0.48

CI, confidence interval; SD, standard deviation.

^a^Proportion of “good” dimension and 95% CI calculated using the Exact Binomial Test.

^b^Cohen’s *d* calculated using the pretest post-test changes in proportions.

^c^Less variability in regularity, a lower Epworth Sleepiness Score, and less time past Midnight represent an improvement in the sleep dimension.

^d^Continuous variable for satisfaction is based on Pittsburgh Sleep Quality Index’s “sleep quality” item with the following responses: 1 (very poor), 2 (fairly poor), 3 (fairly good), and 4 (very good).

^e^Effect size of intervention on sleep efficiency is inflated by the small SDs of the pre- and post-intervention sleep efficiency (2.29% and 2.62%, respectively).

## Discussion

TranS-C, adapted for otherwise healthy adults with suboptimal MDSH and excess weight, was acceptable for the study sample. Given that the data collection approach was appropriate and intervention session engagement was high, a larger study is feasible but would require changes to its recruitment approach. Referrals from primary care providers or sleep specialists could eliminate the need for the preliminary screening decreasing participant burden while also increasing participants’ motivation to complete required study procedures. This could result in enrolling more individuals who could benefit from the intervention.

TranS-C had a large effect on the composite MDSH sleep score and improved 5/6 individual sleep health dimensions. Several prior interventions have attempted to optimize weight loss by improving sleep through educational materials that emphasized the importance of sleep and sleep hygiene principles [14, 15, 25]. These interventions either failed to improve sleep [14, 15] or weight outcomes [15, 25], suggesting that education, alone, is insufficient. Thus, a TranS-C approach, which works through psychosocial, behavioral, and cognitive mechanisms, may be more appropriate.

While all 10 participants who started the intervention completed it, feedback from the semi-structured interviews suggests that the current length of the intervention may be too long. The current length of the intervention (7–8 weeks) could especially be an issue when paired with an existing weight loss intervention. Future research is needed to determine how to balance sufficient session time to build lasting sleep health habits [26] versus engaging in intervention refinement to reduce participant impact (e.g. by reducing the number or length of sessions). Notably, the results should be interpreted with caution, given the small, fairly homogenous sample size and single-arm design. However, the results represent a new approach that addresses MDSH which may serve as a catalyst to improve weight loss and weight loss maintenance. Additional research to examine the long-term effects of the intervention in a large, diverse sample is warranted.

## Data Availability

The data underlying this article will be shared on reasonable request to the corresponding author.
